# Vitamin D Deficiency in Children and Adolescents in Bağcılar, İstanbul

**DOI:** 10.4274/jcrpe.1888

**Published:** 2015-06-03

**Authors:** Meltem Erol, Özgül Yiğit, Suat Hayri Küçük, Özlem Bostan Gayret

**Affiliations:** 1 Bağcılar Training and Research Hospital, Clinic of Pediatrics, İstanbul, Turkey; 2 Bağcılar Training and Research Hospital, Clinic of Biochemistry, İstanbul, Turkey

**Keywords:** adolescents, 25(OH) vitamin D, Vitamin D deficiency, vitamin D insufficiency

## Abstract

**Objective:**

This study aimed to evaluate the frequency of seasonal 25-hydroxyvitamin D [25(OH)D] deficiency and insufficiency in children and adolescents living in Bağcılar, district of İstanbul city.

**Methods:**

Serum vitamin D levels of 280 children aged 3-17 years old were measured at the end of winter and at the end of summer. Of the total group, vitamin D levels were re-measured in 198 subjects. Vitamin D deficiency was defined as a serum 25(OH)D level less than 15 ng/mL and insufficiency-as levels between 15 and 20 ng/mL. Patients whose vitamin D levels were less than 15 ng/mL at the end of winter were treated with 2000 units/day of vitamin D for 3 months.

**Results:**

In the “end of winter” samples, 25(OH)D deficiency was present in 80.36% of the subjects and insufficiency in 11.79%. In the “end of summer” samples, vitamin D deficiency was detected in 3.44% and insufficiency in 27.75%. Vitamin D levels in the “end of winter” samples were not significantly different between boys and girls, while “end of summer” levels were significantly lower in girls (p=0.015). Sunlight exposure was significantly higher in boys (p=0.011). The group with sufficient dairy product consumption had significantly higher vitamin D levels in both “end of summer” and “end of winter” samples. Limb pain was frequently reported in children with low vitamin D levels in the “end of winter” samples (p=0.001). Negative correlations were observed between vitamin D levels and season and also between vitamin D levels and age.

**Conclusion:**

It is essential to provide supplemental vitamin D to children and adolescents to overcome the deficiency seen especially at the end of winter.

## INTRODUCTION

Vitamin D is essential for calcium (Ca) balance and bone tissue mineralization and durability. Its deficiency causes rickets in children and osteomalacia in adults (1). Following the observation in 1920 that vitamin D is effective in treating rickets, western countries started to provide this vitamin as a supplement to infants and children and also to fortify foods such as milk and bread with vitamin D, measures which led to eradication of vitamin D deficiency as a public health problem in these countries (2,3). In developing countries such as Turkey, rickets has persisted as an important childhood illness until recently. Rickets is associated with increased rates of morbidity due to lower respiratory tract infections during infancy (4,5). In recent years, deficiency of vitamin D has become more marked in developed countries and the effects of vitamin D on tissues have gained more importance than its effect on bones. Therefore, a state of “subclinical vitamin D deficiency” was defined and criteria for vitamin D and its deficiency were updated again (6,7,8,9). In our country, vitamin D deficiency has been a significant health problem in children under 3 years old until recently and efforts have been carried out to prevent this deficiency (10). The Ministry of Health has initiated a program envisioning administration of 400 IU vitamin D (3 drops of D vit 3 daily) to all neonates from the first day of their lives and distribute free vitamin D in healthcare centers (10,11). Following this campaign, it was observed that the frequency of rickets in children younger than age 3 years decreased to below 1% (12). In Turkey, studies evaluating the vitamin D deficiency in children older than 3 years are limited (13). Although rickets is seen frequently during infancy and early childhood, due to the rapid growth during the onset of puberty, this period stands out as a second common period for this disorder. With onset of puberty, there is an acceleration in rate of growth and bone mass increases, both leading to an increase in requirements for vitamin D and Ca (14).

In this present study, we evaluated the levels of vitamin D in children older than age 3 years. The evaluation was made at the end of the winter season and the results were compared with levels at the end of the following summer.

## METHODS

The study was conducted in Bağcılar Training and Research Hospital Clinic of Pediatrics in İstanbul. The Ethics Committee of the hospital approved the study protocol (no.2013/148). Children with chronic illnesses, those under regular medical treatment and those with growth failure and/or obesity were excluded from the study. After written informed consent was obtained from the parents, 25-hydroxyvitamin D [25(OH)D] levels of 280 children between 3 and 17 years of age who had applied to our hospital’s pediatric outpatient clinic were analyzed. Of these 280 cases, 134 were boys and 146 were girls. The mean age of the cases was 10.4±2.6 years. Detailed physical examinations were performed to all children. Consumption of milk and dairy products, daily sunlight exposure and the presence of extremity and joint pain were inquired for all children.

Blood samples were taken into biochemistry tubes with gel reagent from the antecubital vein between 08:00-11:30 a.m. for determination of serum 25(OH)D, Ca, inorganic phosphate (P) and alkaline phosphatase (ALP). Blood samples taken during March-April 2012 were assumed to reflect the ‘end of winter’ values, while those taken during November-December 2012 - the ‘end of summer’ levels. We were able to obtain both winter and summer blood samples in 198 of the children. Intact parathyroid hormone (PTH) measurements were also performed in the ‘end of summer’ samples.

Following coagulation, the blood samples were centrifuged at 4000 g for 8 minutes. The serum was transferred into serum separation tubes and kept at -80 oC until analysis. On the day of the analysis, the samples were unfrozen and were measured using an electro-chemiluminescence immunoassay (ECLIA; Roche Cobas 600 immunoassay). Serum Ca (Arsenazo III method), P (Phosphomolybdate/UV method), ALP (DEA method,) levels were measured with ADVIA 1800 analyzer. Intact PTH levels were measured by the direct chemiluminescence method using the ADVIA Centaur XP device of Siemens Diagnostics.

Evaluation of adequacy of diet for provision of Ca was based on the assumptions that 200 mL of milk+1 cup of yogurt and 2 slices of white cheese (800 mg Ca) are adequate for daily Ca intake in children aged 3-8 years and 400 mL of milk+2 cups of yogurt and 3 slices of white cheese (1300 mg Ca) in those aged 9-17 years. Daily sunlight exposure duration was classified as low if it lasted less than 30 minutes and as high if more than 30 minutes. Medical history included asking about presence and if present, frequency of joint and limb pain.

Vitamin D deficiency was defined as a serum 25(OH)D level of <15 ng/mL and insufficiency as a 25(OH)D level between 15 and 20 ng/mL. Vitamin D level >20 ng/mL was accepted as sufficient.

Patients whose vitamin D levels were less than 15 ng/mL at the end of winter were treated with 2000 units/day of vitamin D for 3 months.

### Statistical Analysis

Statistical analysis were conducted using the Number Cruncher Statistical System (NCSS) 2007 Statistical Software (Utah, USA) package. In the evaluation of the data, not only descriptive statistical methods (average, standard frequency and percent distributions) were used but also tests such as unilateral variance analysis in multiple group comparisons, the Tukey’s multiple-comparison test in subgroup comparisons, the independent t-test in binary group comparisons, the matched t-test for ‘end of winter’ and ‘end of summer’ comparisons, the chi-squared test for qualitative data comparisons and the Pearson’s correlation test to determine the relationship of the variables with one another. The results were evaluated using a significance value of p<0.05.

## RESULTS

The population of the Bağcılar region of İstanbul is of low economic and socio-cultural level. The mothers’ level of education is generally low. The clothing styles of the girls included in the study, though not very modern, did not cover all the parts of their bodies. The geographic latitude of İstanbul is 41 degrees and except for a short winter season, it is one of the sunniest cities in Turkey.

**Figure 1:**
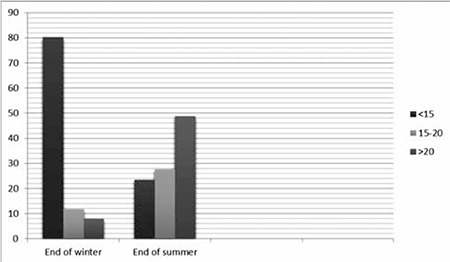
Vitamin D deficiency and insufficiency by season (%).

Analysis of the “end of winter” samples revealed presence of 25(OH)D deficiency in 225 (80.36%) of the children, while insufficiency was present in 33 (11.79%). Only 22 of the children had adequate levels. The results of the “end of summer” showed that 25(OH)D deficiency had regressed to 49 (23.44%), whereas insufficiency had increased to 58 (27.75%) and 102 (48.80%) children had adequate levels (Figure 1). The metabolic characteristics of the study population are given in Table 1.

The “end of summer” mean serum levels of 25(OH)D were significantly higher (p=0.0001) as compared to “end of winter” levels. The mean ages of girls and boys were similar (p=0.697). Also, no significant difference in vitamin D levels at the end of winter levels were noted between girls and boys (p=0.538), while the levels at the end of summer were significantly higher in boys (p=0.015). PTH levels in girls at the end of summer were significantly higher than in boys (p=0.047) (Table 2).

Sufficient sun exposure was significantly less common in girls than boys (p=0.011). No significant gender difference was found in either consumption of milk and dairy products or presence of joint and limp pains (p>0.05). Also, no significant gender difference was observed among those receiving treatment with vitamin D at the end of winter (Table 3).

At the end of summer and at the end of winter, the vitamin D values of the group spending enough time outside were significantly higher compared to those of subjects not spending enough time outside (p=0.0001). Also, the vitamin D values at the end of winter of the group with a good intake of milk and dairy products were found to be statistically significantly higher than those of the group not consuming enough milk and dairy products. At the end of summer, vitamin D levels of the group with a good intake of milk and dairy products and the group with insufficient intake did not show a significant difference (p=0.649). At the end of winter, vitamin D values in the group with complaints of frequent joint pain were found to be significantly lower when compared to the group with no or infrequent extremity complaints (p=0.001, p=0.049). No significant difference was observed between the group with no extremity complaints and the group with infrequent complaints (p=0.864). No significant relationship was found between extremity complaints and vitamin D levels at the end of summer (p=0.464) (Table 4).

A significant negative correlation was found between vitamin D levels at the end of winter and age (r=-0.203 p=0.001). A significant negative correlation was also found between vitamin D values at the end of summer and age (r=-0.184 p=0.008). Age and seasonal distribution of vitamin D levels are given in Table 5.

A significant negative correlation was found between vitamin D levels at the end of summer and PTH levels (r=-0.270 p=0.001). A significant negative correlation was also found between vitamin D values at the end of winter and ALP levels (r=-0.454, p=0.0001). A significant positive correlation was found between vitamin D levels at the end of winter and serum Ca levels (r=0.508, p=0.0001)

## DISCUSSION

Childhood and adolescence are critical periods in terms of skeletal development and bone density. In addition to genotype, physical activity, diet and sufficient vitamin D levels are important factors for reaching optimal bone mass (15,16). The level of vitamin D is affected by many factors such as exposure to the sun, clothing style, skin pigmentation, latitude of region, consumption of dairy products and fish and vitamin supplementation (10,13). Vitamin D status in the human body is assessed by determination of serum 25(OH)D level. The normal levels for serum vitamin D in children and adolescents, as well as in adults, is still a topic of debate (17). Vitamin D status in children is assessed according to the recommendations of the American Pediatric Endocrine Association, which state that 25(OH)D levels between 15 ng/mL and 20 ng/mL should be regarded as insufficiency, levels less than 15 ng/mL as deficiency and levels less than 5 ng/mL as severe deficiency (8). Vitamin D deficiency in childhood and adolescence is an important health problem in Turkey. In this study, “end of winter” blood samples showed a vitamin D deficient state in 80.36% of the cases. Vitamin D insufficiency was present in 11.7% of the cases and a sufficient level was found in only 7.89%. The “end of summer” samples revealed vitamin D deficiency in 23.44% and insufficiency in 27.75%. The proportion of subjects with adequate levels had increased to 48.8%. In previous papers from Turkey, Hatun et al (18) who conducted a study in Kocaeli province (in the same latitude as İstanbul) focused on female adolescents aged 13 to 17 years and found that if the cut-off value for vitamin D deficiency was <25 nmol/L (10 ng/mL) and that for insufficiency was 25-50 nmol/L (10-20 ng/mL), 21.3% of the cases had vitamin D deficiency, while 43.8% had vitamin D insufficiency. Olmez et al (19), in a study conducted in İzmir (latitude 38o), found that the “end of winter” vitamin D insufficiency rate was 59.4%, while that of the “end of summer” was 25%. In a study conducted in Ankara (latitude 39o) among children aged 1 to 16 years, it was reported that the “end of winter” vitamin D insufficiency rate was 25.5%, while the deficiency rate was 8% (13). Karaguzel et al (20) conducted a study in Trabzon (latitude 41o) involving 746 adolescents aged 11 to 18 and found that vitamin D deficiency [25(OH)D <10 ng/mL] frequency was 93% in the spring and 71% in the autumn. Our results are compatible with previous studies. The main reasons for a high frequency of vitamin D insufficiency in a certain location are low vitamin D intake, low sunshine exposure and living at high latitudes. People living below the latitude of 35 degrees are exposed to more sunlight compared to those at higher latitudes (21). However, this does not necessarily ensure optimal vitamin D levels. The best example of this is a study conducted on adolescents in Sao Paulo (latitude 23o), a city close to the equator, which showed a prevalence of vitamin D insufficiency of 60% (22). When adolescent data from National Health and Nutrition Examination Survey (NHANES III) were evaluated, it was found that in the United States, vitamin D insufficiency in winter at low latitudes was 47% in females and 25% in males, while these values were 28% in females and 21% in males at high latitudes in summer months (23). These findings show that season is possibly more important than latitude as a factor affecting vitamin D levels. Accordingly, in our study and in other studies conducted in Turkey, vitamin D levels in “end of winter” samples were found to be markedly lower compared to the “end of summer” samples. For sufficient vitamin D synthesis, the legs and arms should be exposed to the sun between the hours of 10:00 and 15:00 twice a week. The European Society for Paediatric Endocrinology (ESPE) Bone Club states that this period of exposure should be twice a week and highlights that this period can be spent with full clothing but without a hat (24). In Turkey, nearly half of females of adolescent age wear sun-resistant clothing and spend most of their times in closed places (25). In our study, the clothing of the female children was not of the Muslim traditional covering style. In both the “end of winter” and “end of summer” samples, vitamin D levels of the group with more than 30 minutes of daily sun exposure were significantly higher than those of children with less sun exposure. Studies report that the vitamin D levels are lower in female compared to male children (26,27,28,29,30). Adolescent males receive more vitamin D through food compared to females and reach higher serum 25(OH)D levels (31). In our study, the vitamin D levels of female children at the end of summer were significantly lower than those of male children. Moreover, females spent less time outdoors. This may be due to the fact that the region has a low socioeconomic level and for that reason female children spend less time outside. However, no significant relationship was found between gender and vitamin D level in the “end of winter” samples. Vitamin D levels were low in both groups. However, vitamin D levels in the “end of winter” samples of children with low intake of dairy products were found to be significantly low when compared with those with normal intake. This shows that children receive insufficient amounts of vitamin D through nutrition. It is known that Ca and phosphorus consumption of infants and adolescents in Turkey and other Mediterranean countries is low (19,32,33). In the 1920s, when it was realized that vitamin D is important for bone health, supplementation of milk and other dairy products with vitamin D were initiated in many countries. Nonetheless, in infants, small children and adolescents, vitamin D deficiency rates of 12-24% were reported in several countries (16,34). Vitamin D-fortified foods are not common in Turkey. In our study, we believe that factors such as the weather being cold in the winter and the days shorter, air pollution and children spending most of their times indoors explain the low vitamin D level of the children at the end of winter.

There is a relationship between low socioeconomic level and vitamin D insufficiency in adults. In addition, the vitamin D levels of children of mothers with a low education level is also low in infancy and in the adolescent period (35). In a study conducted in two regions of İzmir on subjects of different socioeconomic levels, it was observed that adolescent female children in the group with a low socioeconomic level and whose mothers were of low educational level, had lower vitamin D levels (w). Also, these children were not receiving vitamin D supplementation.

In childhood and adolescence, there is an inverse relationship between PTH and 25(OH)D levels. Thus, the harmful effects of insufficiency of vitamin D on bones are compensated by PTH increase (1,16,36). In our study, in line with previous reports, it was observed that the PTH level in the “end of summer” samples showed an inverse relationship with vitamin D level (16,17,20,29). One of the limitations of our study is that we had no means to assess PTH levels in the “end of winter” samples. The PTH levels of female children in the “end of summer” samples were significantly higher compared to that in male children, while the vitamin D levels of the females were significantly lower.

Starting early in life, frequency of vitamin D deficiency and insufficiency increase with age and becomes more pronounced in adolescence and at older ages. In a study conducted in England, the frequency of vitamin D deficiency was less than 7% among children aged 1.5-10 years; this rate then increased to 11-16% in 11 to 18 years old adolescents. Especially in wintertime, this increase becomes more significant (14). Also in our study, there was a negative correlation between age and both “end of winter” and “end of summer” levels of vitamin D. Vitamin D levels in the adolescent age group were also lower. Absoud et al (37) conducted a study in British children between the ages of 4 and 18 years in which they found that the rate of vitamin D insufficiency was 35%. According to these authors, the risk factors are defined as being adolescent, watching TV for 2.5 hours or more per day, obesity, staying out in the sun less than half an hour and exercising for less than half an hour. A very low vitamin D level can result in symptomatic rickets in adolescents as well. Generalized pain in load-bearing joints such as the vertebrae, femur, calf, difficulty in walking, climbing stairs and running, muscle cramps, facial twitches, generalized weakness, deformity in lower extremities and carpopedal spasms can be observed in these cases (38,39). A number of our cases reported extremity pains. The winter vitamin D level of the group with frequent extremity pain was lower than that in the group without pain. However, active rickets was not detected at physical examination.

Factors such as low socioeconomic and education levels of the families, low intake of vitamin D, air pollution, high latitude and low sun exposure of the children possibly play a role in the development of vitamin D deficiency in our region.

In the event of deficiency according to the vitamin D level measured at the end of winter, we administered 2000 U/day of vitamin D to our cases. However, at the end of summer, the insufficiency was observed to persist, indicating that even when treatment is administered at the end of winter, subclinical deficiency may continue. Weaver and Fleet (40) reported that in female adolescents, a daily intake of 1063 IU vitamin D is necessary to maintain a 32 ng/mL serum vitamin D level.

Holick (1) claimed that in adolescents and adults, an intake of 1500-2000 IU/day of vitamin D could provide sufficient levels in the blood. The ESPE bone club determined the daily vitamin D need of adolescents as 0-1000 IU. It is recommended that the vitamin D level in children and adolescents should be maintained at or over 20 ng/mL and for this reason, that a daily 400 U vitamin D supplementation should be given (41).

In conclusion, this study also showed that vitamin D deficiency and insufficiency in children and adolescents are important problems in the Turkish population. Vitamin D deficiency was prominent in the “end of winter” samples. Despite vitamin D treatment, second samples taken at the end of summer showed persistence of vitamin D insufficiency which may be due to low sunshine exposure, air pollution and low vitamin D intake. Considering the fact that the food and dairy products are not sufficiently fortified with vitamin D in Turkey, vitamin D supplementation is necessary in later childhood and adolescence as in the first years of life.

## Figures and Tables

**Table 1:**
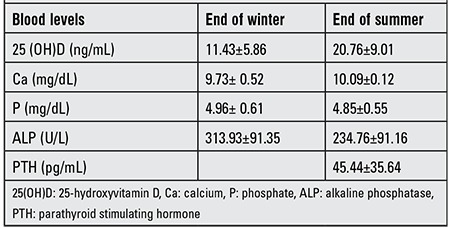
The metabolic characteristics of the study population.

**Table 2:**
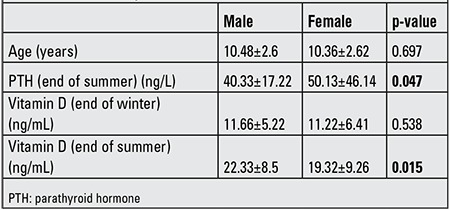
Vitamin D levels at the end of summer and end of winter in male and female subjects.

**Table 3:**
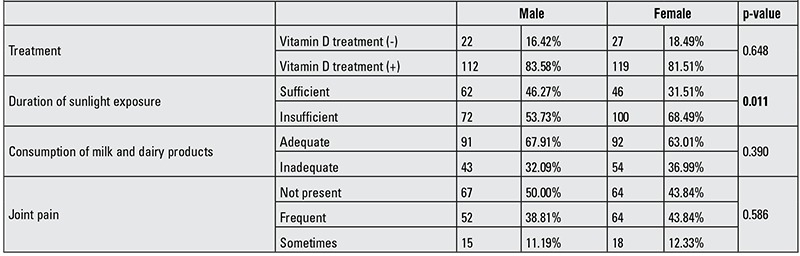
Factors affecting vitamin D level in male and female subjects.

**Table 4:**
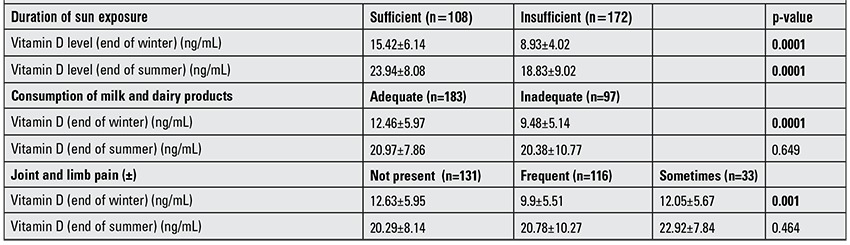
The association of mean vitamin D values with exposure to sunlight, consumption of dairy products and extremity pains.

**Table 5:**
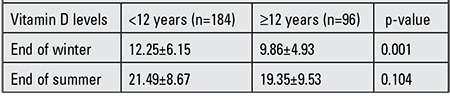
Seasonal distribution of vitamin D level by age group.
